# Surface plasmon enhanced Organic color image sensor with Ag nanoparticles coated with silicon oxynitride

**DOI:** 10.1038/s41598-019-57087-2

**Published:** 2020-01-14

**Authors:** Sung Heo, Jooho lee, Gae Hwang Lee, Chul-Joon Heo, Seong Heon Kim, Dong-Jin Yun, Jong-Bong Park, Kihong Kim, Yongsung Kim, Dongwook Lee, Gyeong-Su Park, Hoon Young Cho, Taeho Shin, Sung Young Yun, Sunghan Kim, Yong Wan Jin, Kyung-Bae Park

**Affiliations:** 1 Platform Technology Lab, Samsung Advanced Institute of Technology, 130, Samsung-ro, Yeongtong-gu, Suwon-si, Gyeonggi-do 443-803 Republic of Korea; 2 Organic Materials Laboratory, Samsung Advanced Institute of Technology, 130, Samsung-ro, Yeongtong-gu, Suwon-si, Gyeonggi-do 443-803 Republic of Korea; 3 Department of Physics, Dongguk University, Seoul, 04620 Korea; 4 Department of Physics, Yonsei University, 1 Yonseidae-gil, Wonju-si, Gangwon-do 26493 Republic of Korea; 5 Department of Materials Science and Engineering, Seoul National University, Seoul, 08826 Republic of Korea; 6 Department of Chemistry, Chonbuk National University, Jeonju, 54896 Republic of Korea

**Keywords:** Sensors and biosensors, Characterization and analytical techniques, Nanoparticles

## Abstract

As organic photodetectors with less than 1 *μ*m pixel size are in demand, a new way of enhancing the sensitivity of the photodetectors is required to compensate for its degradation due to the reduction in pixel size. Here, we used Ag nanoparticles coated with SiO_x_N_y_ as a light-absorbing layer to realize the scale-down of the pixel size without the loss of sensitivity. The surface plasmon resonance appeared at the interface between Ag nanoparticles and SiO_x_N_y_. The plasmon resonance endowed the organic photodetector with boosted photon absorption and external quantum efficiency. As the Ag nanoparticles with SiO_x_N_y_ are easily deposited on ITO/SiO_2_, it can be adapted into various organic color image sensors. The plasmon-supported organic photodetector is a promising solution for realizing color image sensors with high resolution below 1 *μ*m.

## Introduction

Capturing color images has been a basic human desire since time immemorial. Painting portraits and/or sceneries was the only way of capturing the reflected images of people or things colorfully before Becquerel’s photographic discovery with silver halide in 1848^1^. The introduction of charge-coupled device (CCD)-based image sensors in markets has rapidly replaced film-based photography, and now color image sensors (CISs) are being widely used in imaging electronics such as digital cameras, smart phones, portable computers, and vehicles^2^. Research focus on the resolution of the CIS has increased greatly, and much effort has been made to scale down the size of imaging pixels on a chip with Si technology. As a front leader in CISs, Si-based complementary metal–oxide–semiconductor (CMOS) image sensors^3–5^, which are made up of Bayer-patterned color filter arrays^6^, have made impressive progress in enhancing the image sensor performance, and have experienced commercial success even after the introduction of stacked structures. However, they are faced with a great challenge to realize high resolutions without any loss of sensitivity as the pixel size has scale-downed below 1 *μ*m. Although the integration of CMOS with plasmonic color filters boosted sensitivity owing to increased transmission and the adoption of nanowires for filter arrays further improved the sensitivity^7–9^, they are not fundamental solutions to the problems caused by the low photon absorption coefficient of Si and the limited light-receiving area of pixels. Thus, researchers have been searching for an alternative to Si-based CMOS. Meanwhile, the advent of organic photodiodes has expanded the CIS domain further to flexible and color filter-free image capturing electronics^10–12^. As a hybrid-type, organic-on-Si-CMOS image sensors with a stacked structure^13–15^, where the top photo-conversion layer serves as a photodiode for green light and the bottom layer works as photodiode for blue and red lights, have doubled the light-receiving area owing to the stacked layers where light is absorbed. Accordingly, the number of photons absorbed/used for charge carrier creation in the sensors becomes doubled. Furthermore, a higher spatial resolution is achieved with minimized color Moiré noise. However, organic photodetectors (OPDs) are now confronted with big challenges. First, new materials with narrowband and high absorption are in urgent need. Second, noise occurring from non-negligible absorption in unwanted regions should be minimized and the linear dynamic range should be improved. In this study, we are focused on controlling photon absorption and external quantum efficiency (EQE) using surface plasmon effect to realize high resolution with low noise.

Among the basic collective modes for improving photon absorption, surface plasmon-polariton has been investigated in various fields such as solar cell^16–18^, organic light-emitting diodes (OLEDs)^19–22^, and 2D materials^23–26^ for improving photon absorption. Recently, it has been adapted in solar cells and OLEDs to enhance their efficiencies^17,18^. Moreover, light absorption, which is a vital factor for EQE, has been increased in OPD with metal layers by plasmon resonance effect^27–29^. Metal nanoparticles (NPs) such as silver and gold have been also used to increase the EQE and signal-to-noise ratio (SNR) via surface plasmon resonance (SPR)^30–34^. NPs in the active layer in OPDs absorb the incident photon and plasmon-assisted NPs absorb more photon when a polariton is created. The coupling between the plasmon and NPs depends on the size and shape of NPs. As the size of the NPs becomes smaller than the wavelength of light, a surface plasmon is confined in NPs, which is called a “localized surface plasmon resonance (LSPR)”^26,35–41^. The LSPR has two main advantages: the electric field enhancement at the metal surface and the maximum optical absorption at the plasmon resonance frequency. The first accelerates the generation of excitons, while the second promotes the absorption of photons. Moreover, the coupling between the plasmon and incident photon is also influenced by the materials surrounding metal nanoparticles, i.e. the plasmon frequency may be tuned by the surrounding material. Therefore, the EQE can be improved along with the plasmon resonance if proper surrounding materials and NPs are chosen. Here we report that photon absorption in the OPD was surged by LSPR in Ag NPs coated with SiO_x_N_y_ and the EQE was also improved.

**Figure 1:**
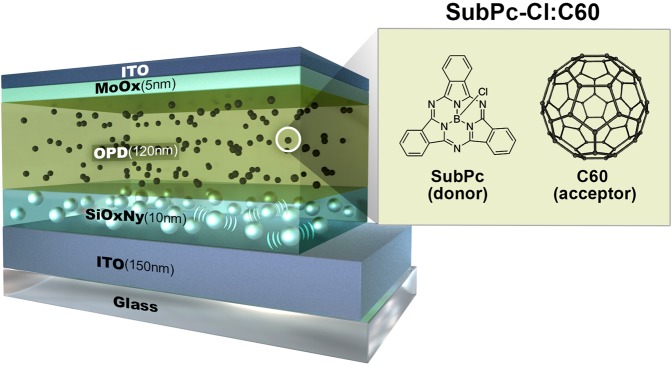
Structure of the OCIS with Ag/SiO_x_N_y_ buffer layer. Glass substrate/ITO(150 nm)/Ag nanoparticles SiO_x_N_y_ (10 nm)/blend layer(120 nm)/ITO. The blend layer is composed of C60 (acceptor) and Boron subphthalocyanine chloride (SubPc-Cl, donor).

Figure 1 illustrates the structure of the OPD device. The SubPc-Cl:C60 is an organic semiconducting push-pull molecule, of which absorption band lies in the green-light region and energy level difference between the highest occupied molecular orbital (HOMO) and lowest unoccupied molecular orbital (LUMO) is 1.9 eV. The photon absorption enhancement in this study was investigated with the structure shown in Fig. 1: ITO/Ag/SiO_x_N_y_/blend layer//ITO.

## Results and Discussion

**Figure 2:**
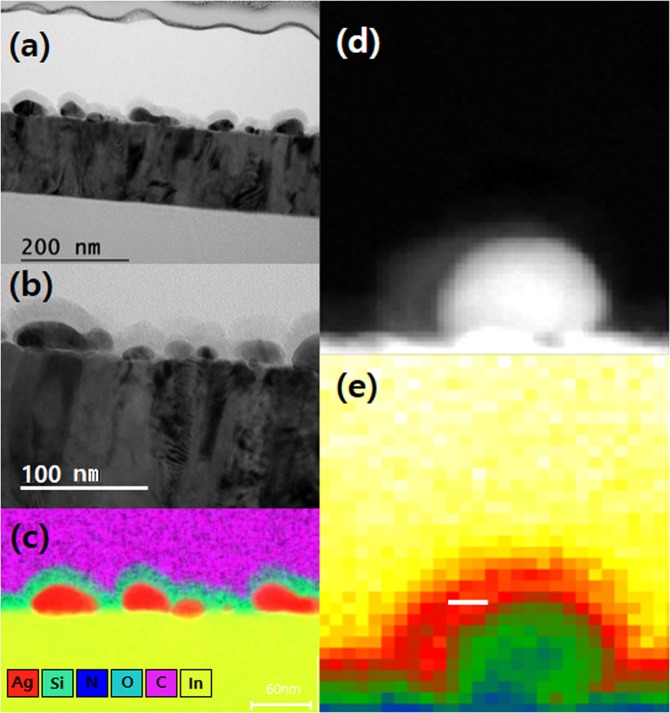
(**a**,**b**) Cross-sectional TEM images of the OPD. (**c**) EDX mapping of the enlarged region in (**b**). Ag NPs (Red in color) coated with SiO_x_N_y_ are distinctly visible. (**d**) Enlarged TEM image of Ag NP coated with SiO_x_N_y_. (**e**) EELS mapping image superimposed on a TEM image.

Figure 2(a) illustrates the cross-sectional transmission electron microscopy (TEM) image of the OPD device. Ag NPs coated with SiO_x_N_y_ are clearly visible in Fig. 2(c). Electron energy loss spectroscopy (EELS) mapping is superimposed on a TEM image in Fig. 2(e), where the mark denoted by a white line implies that a localized surface plasmon is formed on that mark and the energy is in the order of 2.4–2.6 eV. The non-retarded surface plasmon condition for the metal-dielectric interface is ε_1_ + ε_2_ = 0, where ε_1_ and ε_2_ are permittivities for each material. Since the permittivity of Ag at 543 nm for green light absorption is −10.2^42^, the amorphous silicon oxynitride film surrounding Ag NPs should have the permittivity of 9.8. To investigate the permittivities of Ag NPs and SiO_x_N_y_ layer, EELS measurement with the help of *Kramers-Kronig* relation was carried out.

**Figure 3:**
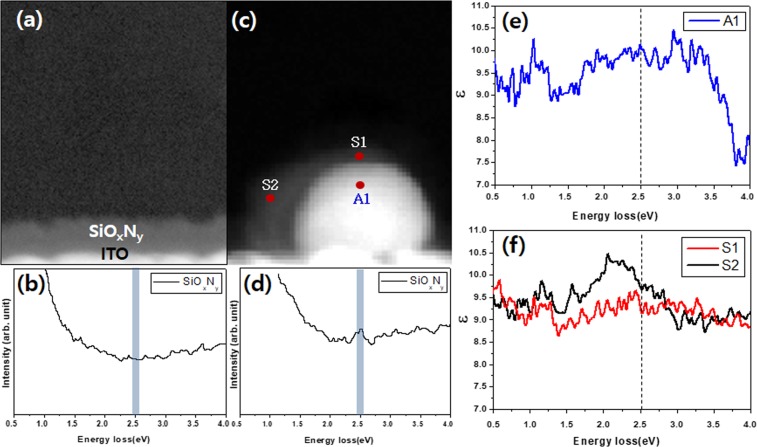
TEM-EELS measurements for the permittivities of Ag NP and SiO_x_N_y_. (**a**) TEM of SiO_x_N_y_ layer on ITO (**b**) EELS spectrum of the SiO_x_N_y_ layer in (**a**). (**c**) TEM image of Ag NP coated with SiO_x_N_y_. (**d**) EELS spectrum of SiO_x_N_y_ layer in (**c**,**e**) Permittivity measurement by TEM of Ag (A1). (**f**) Permittivity measurement by TEM of SiO_x_N_y_ on Ag nanoparticle (S1 and S2).

The direct investigation of the surface plasmon in the OPDs was carried out by TEM-EELS and the results are shown in Fig. 3. Figure 3(a) shows the image of SiO_x_N_y_ on ITO. Figure 3(b) displays no distinct peak from the conduction band of SiO_x_N_y_, showing no sign of extra elementary excitation mode. In Fig. 3(c), Ag NPs coated with SiO_x_N_y_ are clearly visible. Free electrons in Ag NPs move the surface and they are accumulated on the surface. The electron density is very high and several electric dipoles are formed at the interface between SiO_x_N_y_ and Ag NPs. The electric dipole is strong enough for the excitation of the surface plasmon. The formation of the localized surface plasmon in Ag NP coated with SiO_x_N_y_ accelerates the absorption of the incident photons. The permittivities of Ag NP and SiO_x_N_y_ in Fig. 3(c) were measured and were illustrated in Figs. 2(f) and 3(e). The absolute value of the permittivity of Ag NP is 10.25, while SiO_x_N_y_ has a range of permittivities from 9.39 (S1) to 9.75 (S2). Therefore, the surface plasmon appears in the region where the sum of the permittivities of Ag NP and SiO_x_N_y_ is zero.

**Figure 4:**
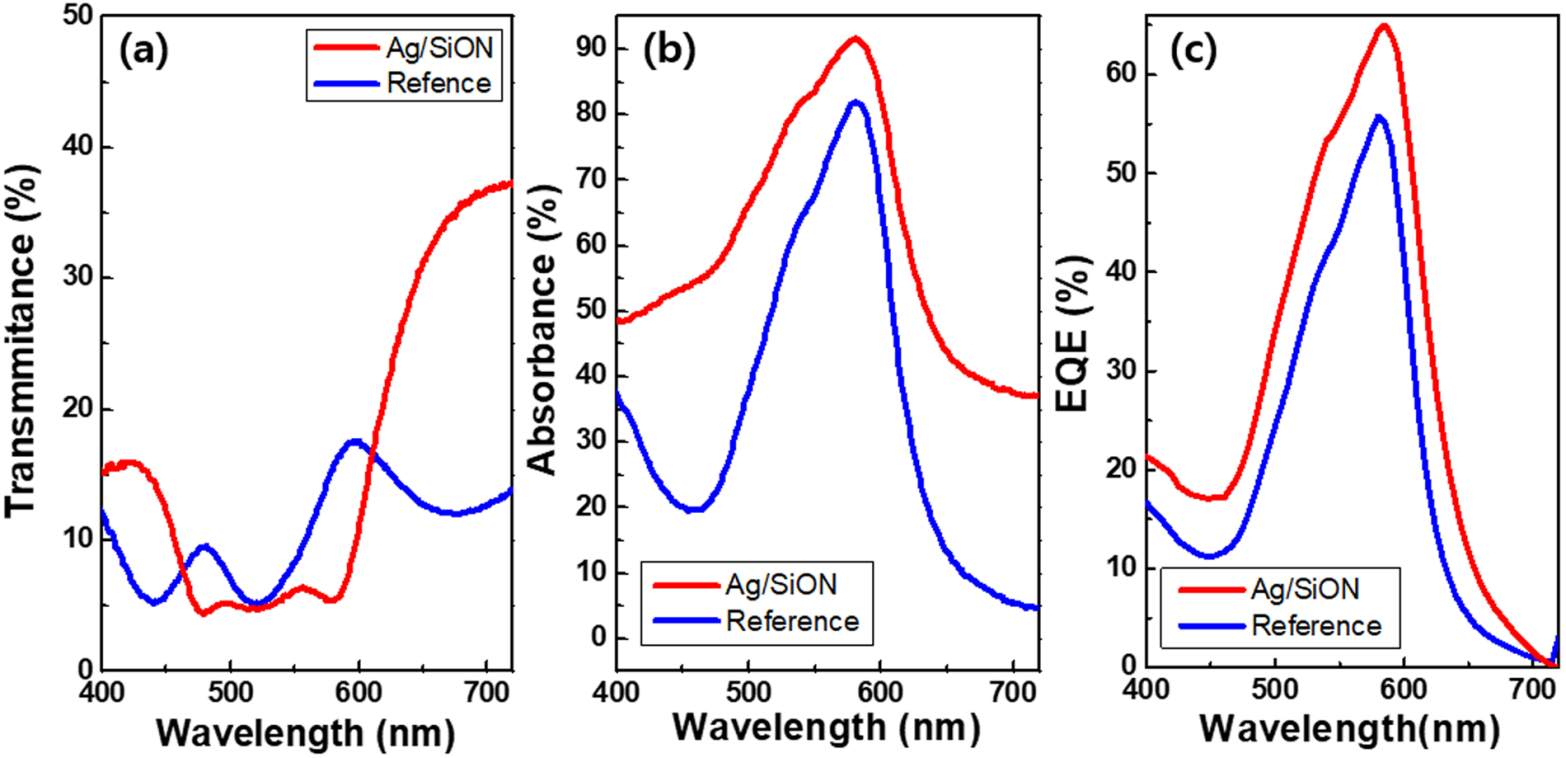
Optical properties of the reference sample and the OPD. (**a**) Transmittance curves. (**b**) Absorbance curves. (**c**) EQE curves.

The transmittances of the OPDs are compared in Fig. 4(a). In the green wavelength range of 460 nm to 590 nm, the reference OPD does not show uniform transmittance and the transmittance goes very high near 600 nm, while the transmittance is the lowest at 520 nm. Meanwhile, the Ag/SiO_x_N_y_ OPD shows uniform transmittance in the green wavelength range. If the transmittance and absorbance curves are like a rectangular function, the color selectivity and color uniformity will be enhanced. However, in real life, the transmittance and absorbance curves are different from a rectangular function. On the contrary, in several cases, they show a peaky or triangular shape, implying that the OPD becomes sensitive only at about 520 nm centered in the green wavelength and less sensitive at about 480 nm and 590 nm.

Figure 4(b) shows the absorbance spectra of the OPD devices. The blue line is from the conventional OPD, illustrating narrow bandwidth of 80 nm centered at 526 nm, while the red line comes from the OPD with Ag/SiO_x_N_y_ which exhibits a slightly broad bandwidth of ~120 nm centered at 524 nm. Compared with the absorption of the conventional OPD, the Ag/ SiO_x_N_y_ OPD displays 8% higher absorption (85–>93%). The absorption difference is caused by the plasmon effect in Ag/SiO_x_N_y_. The Ag/SiO_x_N_y_ OPDs also display much more improved EQE characteristic with a maximum of 65% at 580 nm, while the conventional OPDs have 55% of the EQE.

**Figure 5:**
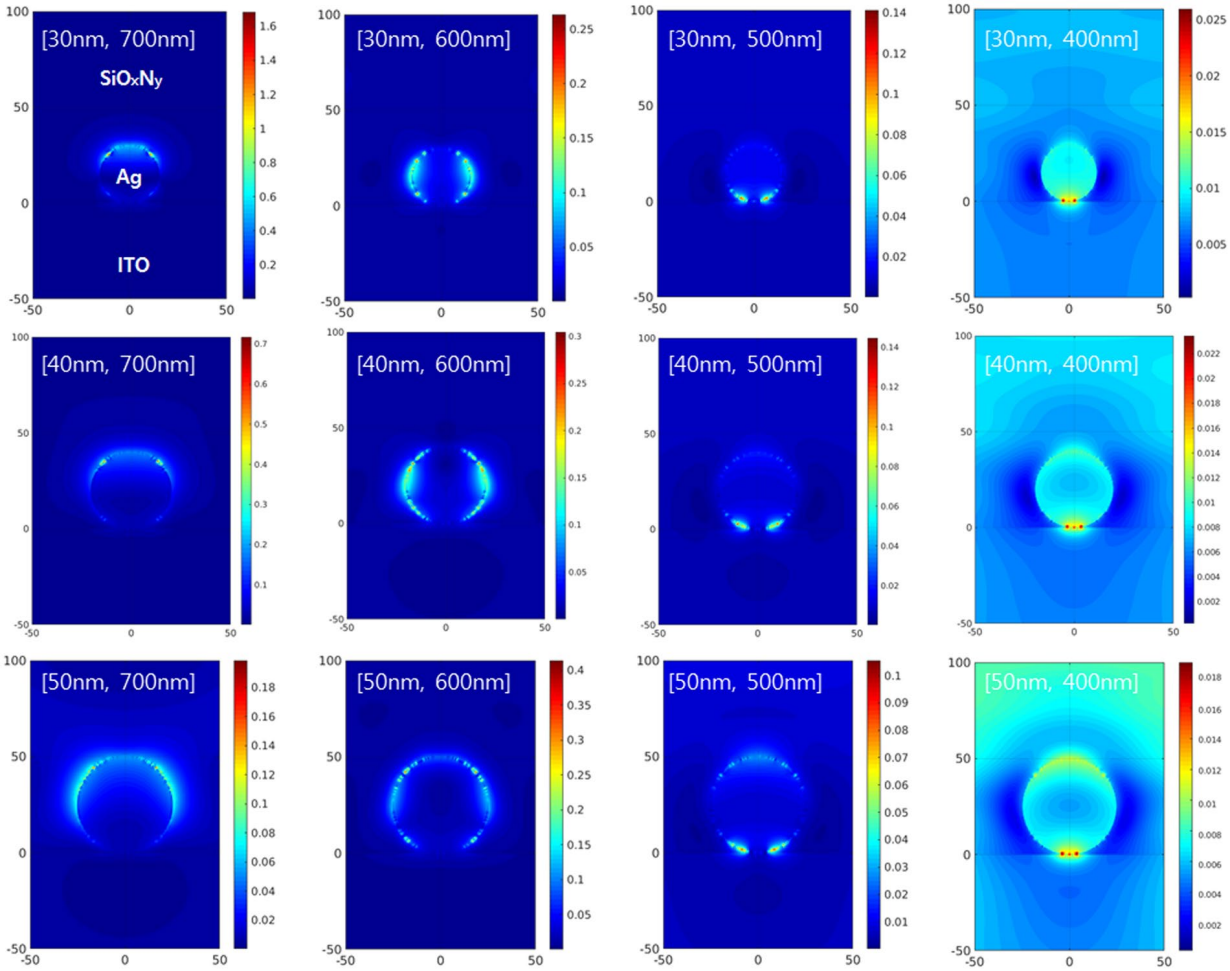
Simulated electric field enhancement patterns of Ag NPs (diameters 30 nm, 40 nm, and 50 nm) coated with SiO_x_N_y_ on ITO substrate at four optical wavelengths (400 nm, 500 nm, 600 nm, and 700 nm), displaying LSPR images produced by the collective oscillations of the free electrons.

To understand the role of Ag NPs in the OCIS for absorption enhancement, the excitation of LSPR as a function of wavelength of the incident photon with varying diameters of Ag-NP coated with SiO_x_N_y_ was simulated with the finite difference time domain (FDTD) method and the results are shown in Fig. 5. When UV is incident on Ag NP, the excitation of LSPR is negligible and slightly appears at the interface between Ag NP and the substrate (ITO). With the incidence of 500 nm photons, the LSPR modes still remain near the interface between the Ag NP and the substrate. However, the strong excitation of the LSPR spreads along the surface of the Ag NP when 600 nm photons are incident. When the wavelength of the incident photon is 700 nm, the LSPR modes are excited on the upper surface.

Generally, the light intensity of a localized surface plasmon at metal nanoparticles/insulator interface is proportional to that inside the metal nanoparticles, i.e. the stronger the LSPR is formed at the interface, the more the light is absorbed and confined inside the metal nanoparticles. In Fig. 5, the excitation of LSPR creates strong electric fields and evanescently-propagating waves contributing to the absorption enhancement. However, all the wavelengths in Fig. 5 do not equally participate in the increase of photon absorption although they exhibit strong electric field resonances. Below 40 nm size of Ag NP, 600 nm wavelength is the optimum for the excitation of the LSPR, while Ag NPs with a size of 50 nm are most likely to contribute to the absorption enhancement at 700 nm wavelength.

**Figure 6:**
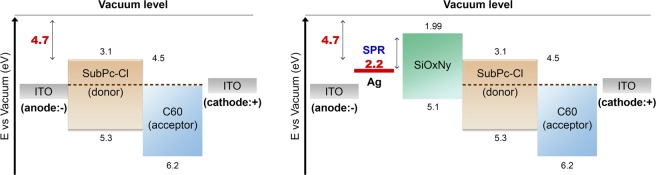
Band structures of the reference OPD and OPD with Ag NP/SiO_x_N_y_ layer. (**a**) The reference OPD (**b**) OPD with Ag NP/SiO_x_N_y_ layer.

Band structures of the reference OPD and the OPD with Ag NP coated with SiO_x_N_y_ layer are constructed and shown in Fig. 6. Both carriers of electrons and holes flow from the ITO anode and cathode to the acceptor and donor with negative reverse bias for the reference. The origin of the leakage current under reverse bias is due to electrons rather than holes because the barrier for electrons between the work-function of ITO (4.7 eV) and the LUMO of C60 (4.5 eV) is 0.4 eV, which is considerably smaller than the hole barrier which is 0.6 eV between the work function of ITO (4.7 eV) and the HOMO level of SubPc-Cl (5.3 eV). However, the SiO_x_N_y_ film in the OPD acts as electron blocking layer (EBL) since the LUMO level of SiO_x_N_y_ is higher than that of SubPc-Cl:C60(1:1) layer. Once Ag NPs coated with SiO_x_N_y_ are inserted between ITO and SubPc-Cl, the barrier for electrons becomes higher and prevents the electrons from transporting from ITO to the acceptor layer, reducing the leakage current. However, when SPR appears at the interface between Ag and SiO_x_N_y_, it generates electrons and holes. SPR-assisted electrons occupy the plasmon energy states, while holes stay near the Fermi level of Ag. Therefore, the SPR facilitates the flow of electrons to the cathode, indicating the increase in the leakage current. In addition, SPR-generated holes staying near the Fermi level of Ag also promote the hole-current toward the anode. Nonetheless, the photon absorption by the LSPR outnumbers the leakage current, causing the leap in the EQE.

**Figure 7:**
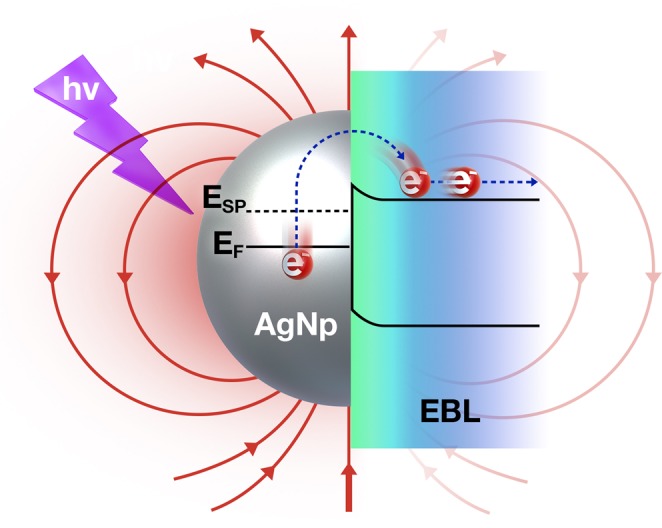
Mechanism of photon-absorption enhancement by surface plasmon resonance.

Figure 7 illustrates the incident photon absorption enhancement by Ag NP coated with SiO_x_N_y_. The energy of the SPR in Fig. 3 measured by TEM-EELS is around 2.2 eV. The excited electrons by SPR have higher energy by 2.2 eV than the Fermi level of Ag. Since the energy difference between the plasmon state and the conduction band of SiO_x_N_y_ is 2.3 eV, the excited electrons by SPR can transfer to the exciton states in SiO_x_N_y_ layer and they can easily arrive at the cathode electrode (ITO) under illumination.

## Conclusion

In summary, the OPD with Ag NPs coated with SiO_x_N_y_ was fabricated. The SPR formed at Ag NPs coated with SiO_x_N_y_ accelerated the photon absorption and improved the EQE. The SPR was investigated and visualized by TEM-EELS. The formation of the SPR at the interface of Ag NP and SiO_x_N_y_ was experimentally confirmed. Although the effective area for receiving the incident photon is expected to decrease with the scaling-down of the pixels, the introduction of the SPR in OCIS counters the problem without losing the spatial resolution. With further systematic research conducted on the pattern and size of Ag NPs, the SPR is likely to be the sole solution for realizing OCISs with high resolution below 1 *μ*m.

## Methods

### Device fabrication

The deposition of the SiO_x_N_y_ buffer layer on ITO glass was sequentially carried out at 180 °C by Plasma-Enhanced Chemical Vapor Deposition (PECVD) using various SiH_4_:NH_3_:NO_2_ gas mixtures with carrier N_2_ gas. The thickness of the SiO_x_N_y_ layer was 10 nm. The ratios of x (O/Si) and y (N/Si) were 0.16 and 0.66, respectively. The OPD layer, the organic blend layer of SubPc-Cl and C60 was deposited. Lastly, the capping layer of ITO was deposited, as shown in Fig. 1.

### Characterization

Compositional analysis was performed by reflection electron energy loss spectroscopy (REELS) using auger electron spectroscopy (AES, PHI-4700, Concentric hemispherical analyzer) and X-ray photoelectron spectroscopy (XPS, PHI Quantera II Scanning XPS Microprobe), respectively. REELS spectra were collected using the primary electron energy of 1.5 eV for excitation and constant analyzer pass energy of 10 eV. The full width at half maximum (FWHM) of the elastic peak was 0.8 eV. TEM-EELS characterization of Ag NPs was conducted using a high-resolution transmission electron microscopy (TEM, Titan 20-200ST, FEI).

## Supplementary information

Supplementary information.
