# Author Correction: Surface plasmon enhanced Organic color image sensor with Ag nanoparticles coated with silicon oxynitride

**DOI:** 10.1038/s41598-020-59906-3

**Published:** 2020-02-13

**Authors:** Sung Heo, Jooho Lee, Gae Hwang Lee, Chul-Joon Heo, Seong Heon Kim, Dong-Jin Yun, Jong-Bong Park, Kihong Kim, Yongsung Kim, Dongwook Lee, Gyeong-Su Park, Hoon Young Cho, Taeho Shin, Sung Young Yun, Sunghan Kim, Yong Wan Jin, Kyung-Bae Park

**Affiliations:** 1 Platform Technology Lab, Samsung Advanced Institute of Technology, 130, Samsung-ro, Yeongtong-gu, Suwon-si, Gyeonggi-do 443-803 Republic of Korea; 2 Organic Materials Laboratory, Samsung Advanced Institute of Technology, 130, Samsung-ro, Yeongtong-gu, Suwon-si, Gyeonggi-do 443-803 Republic of Korea; 3 Department of Physics, Dongguk University, Seoul, 04620 Korea; 4 Department of Physics, Yonsei University, 1 Yonseidae-gil, Wonju-si, Gangwon-do 26493 Republic of Korea; 5 Department of Materials Science and Engineering, Seoul National University, Seoul, 08826 Republic of Korea; 6 Department of Chemistry, Jeonbuk National University, Jeonju, 54896 Republic of Korea

Correction to: *Scientific Reports* 10.1038/s41598-019-57087-2, published online 14 January 2020

The original version of this Article contained a typographical error in the spelling of the author Jooho Lee, which was incorrectly given as Jooho lee.

In addition, the original version of this Article contained an error in Affiliation 6, which was incorrectly given as ‘Department of Chemistry, Chonbuk National University, Jeonju, 54896, Republic of Korea’. The correct affiliation is listed below:

Department of Chemistry, Jeonbuk National University, Jeonju, 54896, Republic of Korea.

These errors have now been corrected in the PDF and HTML versions of the Article, and in the accompanying Supplementary Information file.

