# Supplementary material for: Surface plasmon enhanced Organic color image sensor with Ag nanoparticles coated with silicon oxynitride
Source: Sci Rep. 2020 Jan 14;10:219. doi: 10.1038/s41598-019-57087-2 (PMC6959276; doi:10.1038/s41598-019-57087-2)
Supplement: Supplementary file 1 — Supplementary information. [file 41598_2019_57087_MOESM1_ESM.docx]

**Surface plasmon enhanced Organic color image sensor with Ag nanoparticles coated with silicon oxynitride**

Sung Heo^1,‡^, Jooho lee^1,‡^, Gae Hwang Lee^2^, Chul-Joon Heo^2^, Seong Heon Kim^1^, Dong-Jin Yun^1^, Jong-Bong Park^1^, Kihong Kim^1^, Yongsung Kim^1^, Hoon Young Cho^3^, Dongwook Lee^1,4,*^, Gyeong-Su Park^5^, Taeho Shin^6^, Sung Young Yun^2^, Sunghan Kim^2^, Yong Wan Jin^2^, Kyung-Bae Park^2,*^

^1^ *Platform Technology Lab, Samsung Advanced Institute of Technology, 130, Samsung-ro, Yeongtong-gu, Suwon-si, Gyeonggi-do, Republic of Korea, 443-803.*

^2^ *Organic Materials Laboratory, Samsung Advanced Institute of Technology, 130, Samsung-ro, Yeongtong-gu, Suwon-si, Gyeonggi-do, Republic of Korea, 443-803*

^3^ *Department of Physics, Dongguk University, Seoul, 04620, Korea*

^4^ *Department of Physics, Yonsei University, 1 Yonseidae-gil, Wonju-si, Gangwon-do 26493, Republic of Korea.*

^5^ *Department of Materials Science and Engineering, Seoul National University, Seoul, 08826, Republic of Korea.*

^6^ *Department of Chemistry, Chonbuk National University, Jeonju, 54896, Republic of Korea.*

^†^ Sung Heo and Jooho lee contributed equally to this study

*E-mail: [dongwookleedl324@gmail.com](mailto:dongwookleedl324@gmail.com)

*E-mail : [myshkin.park@samsung.com](mailto:myshkin.park@samsung.com)


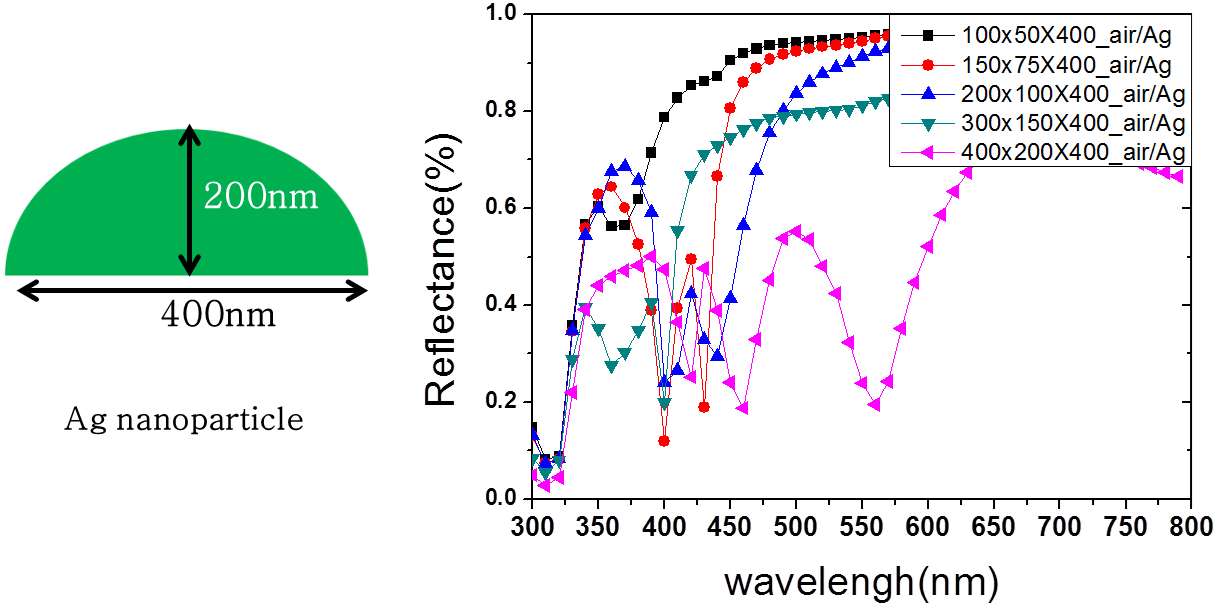


Figure S1. Simulation of the reflectance curves of Ag NPs with various sizes.

Figure S1 shows the reflectance vs the size of Ag NPs. The reflectance depends on the size of Ag NPs. Since the plasmon frequency of Ag NPs is influenced by the shape and the size of Ag NPs, the frequency is tunable.


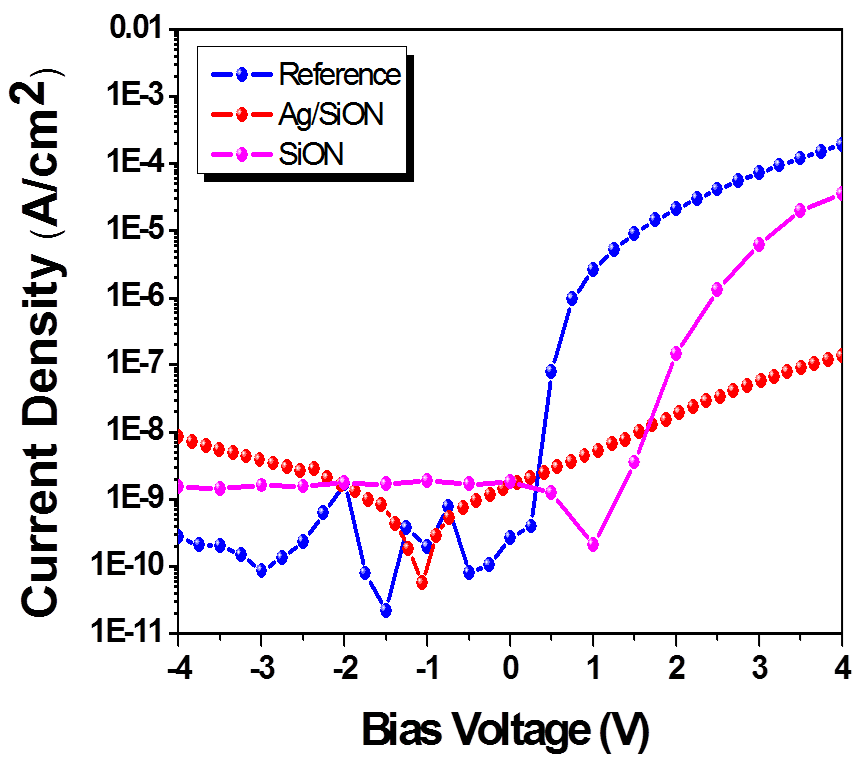


Figure S2. DC characteristics of the OPDs

The Dark Current DC) characteristics of the ITO/Ag/ SiO_x_N_y_ /ITO were compared with that of ITO in Figure 3(b). The DC of the ITO/Ag/ SiO_x_N_y_ /ITO was 3.84 nA/cm2 at -3V, while the ITO has 0.0855nA/cm2 of DC at -3V. The DC characteristics of the OPD with Ag NPs/ SiO_x_N_y_ were not improved, compared with the reference sample.


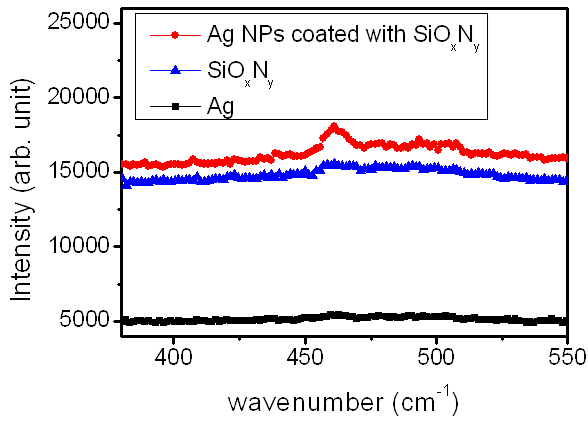


Figure S3. Raman spectra of Ag NPs, SiO_x_N_y_, and Ag NPs coated with SiO_x_N_y_.

Figure S3 compares the Raman spectra of Ag NPs, SiO_x_N_y_, and Ag NPs coated with SiO_x_N_y_. Ag NPs do not show any Raman peak, while SiO_x_N_y_ displays a tiny peak at 460cm^-1^. The Raman peak from Ag NPs coated with SiO_x_N_y_ is enhanced in Figure S3. The enhancement of the peak intensity is caused by Surface enhanced Raman Scattering (SERS), which takes place at the metal surface with the help of surface plasmon and is a strong support for surface plasmon resonance in Ag NPs coated with SiOxNy.
